# Surgical Staging of Differentiated Endometrial Cancer: An Analysis of Postoperative Outcome in a Gynecological Cancer Center in Sri Lanka

**DOI:** 10.7759/cureus.41605

**Published:** 2023-07-09

**Authors:** Rajitha D Wijesinghe, Malitha Patabendige, Nishanthi Pakthagunanathan, Chintana Hapuachchige

**Affiliations:** 1 Gynecological Oncology, Teaching Hospital Mahamodara, Galle, LKA; 2 Obstetrics and Gynaecology, Base Hospital, Mahaoya, Mahaoya, LKA; 3 Gynecological Oncology, Apeksha Hospital, Maharagama, LKA

**Keywords:** grade 1, well differentiated, endometrioid, surgical staging, sri lanka, lymphadenectomy, lymph node metastasis, pathology, histology, endometrial cancer

## Abstract

Introduction: Even though surgico-pathological staging is recommended in poorly differentiated endometrial cancer, management of differentiated endometrial cancer is controversial. Preoperative pelvic and abdominal Magnetic Resonance Imaging (MRI) is recommended in well-differentiated endometrial cancer to identify patients with risk factors for regional metastasis. However, access to MRI is limited in Sri Lanka, and surgico-pathological staging is the primary staging method available for most patients with differentiated endometrial cancer. Our objective was to evaluate the outcome of surgical staging among differentiated endometrial cancer patients who underwent primary surgery at the gynecological cancer center of Apeksha Hospital Maharagama, Sri Lanka.

Methods: A retrospective study was conducted using the ongoing electronic database at the gynecological cancer center of the National Cancer Institute (Apeksha Hospital) in Maharagama, Sri Lanka. Data from December 2019 to December 2020 were selected for analysis.

Results: During the study period, 112 patients with endometrial cancer underwent hysterectomy. This study included 90 patients with differentiated endometrial cancer (International Federation of Gynecology and Obstetrics [FIGO] Grade 1 and Grade 2), out of which pelvic lymph node dissection was performed in 78 (86.7%) cases. Among the 90 patients, 54 (60%) had medical comorbidities. It was reported that 35% (n=32) of the patients had myometrial invasion of more than 50% thickness. Furthermore, 13.8% of patients with deep myometrial invasion had lymph node metastasis, while only one patient (2%) in the superficial or no myometrial invasion group had lymph node metastasis. Therefore, the absence of deep myometrial invasion has a negative predictive value of around 98% for excluding pelvic lymph node metastasis.

Conclusion: Approximately one in seven patients with deeply infiltrating differentiated endometrial cancer had lymph node metastasis. In limited resource settings where preoperative pelvic MRI is not readily available, implementing a policy of routine surgical pelvic lymph node assessment would be beneficial. This approach would aid in detecting stage IIIc disease and also help avoid unnecessary pelvic irradiation.

## Introduction

While endometrial cancer has surpassed cervical cancer as the commonest gynecological cancer in the developed world, its incidence is rising at an alarming rate in low- and middle-income countries [1,2]. Although the management of endometrial cancer is generally considered less challenging compared to other gynecological malignancies, there are still controversies surrounding its treatment. Staging plays a crucial role in the management of any cancer [3]. Accurate staging helps identify patients with advanced disease who may require adjuvant treatment to control distant metastasis. However, surgical staging involving pelvic/para-aortic lymphadenectomy and omentectomy carries additional morbidity. Therefore, surgical staging is tailor-made to each patient according to risk profile for lymphatic and peritoneal metastasis. While it is well-accepted for high-risk patients (poorly differentiated histology, deep myometrial invasion) to undergo comprehensive surgical staging, the best approach for low-risk patients has been subjected to much debate. Preoperative pelvic Magnetic Resonance Imaging (MRI) has been recommended, even in cases of well-differentiated endometrial cancer, to identify patients with deep myometrial invasion and cervical involvement [3-5]. This is particularly relevant when considering the possibility of avoiding pelvic lymphadenectomy [6,7]. However, access to MRI scanning in Sri Lanka faces significant limitations [8]. Consequently, pelvic lymphadenectomy remains the primary staging method available for the majority of patients with differentiated endometrial cancer in Sri Lanka.

This study aimed to evaluate histology outcomes and regional metastasis of differentiated endometrioid endometrial cancer (International Federation of Gynecology and Obstetrics [FIGO] Grade 1 and Grade 2) in patients who underwent surgery at the National Cancer Institute (Apeksha Hospital) Maharagama, the main referral center for gynecological cancer patients in Sri Lanka.

## Materials and methods

A retrospective descriptive study was conducted using the ongoing electronic database at the gynecological cancer center, Apeksha Hospital Maharagama, Sri Lanka. This database contained information on patients' preoperative comorbidities, details of primary surgery and post-operative histology. However, it did not contain data on subsequent follow-up and outcome. Ethical clearance was obtained from the ethics review committee of the Castle Street Hospital for Women - Colombo, Sri Lanka (approval number - ERC/284/02/2021).

Our study population involved patients with endometrial cancer who were referred to the gynecological cancer center of Apeksha Hospital Maharagama from all parts of Sri Lanka for primary surgical management. Apeksha Hospital is the only center for gynecological cancer patients which receives direct referrals from all parts of the country. In addition, there are two other regional gynecological oncology centers that operate in the Southern and Central provinces of Sri Lanka. 

Patients who underwent hysterectomy for endometrial cancer from December 2019 to December 2020 were selected. Metabolic risk factors, surgery details and post-operative histology were extracted from patients who had FIGO Grade 1 and FIGO Grade 2 endometrioid type endometrial cancer.

Metabolic risk factors included diabetes mellitus, hypertension and dyslipidemia. However, BMI was not reported in the electronic database. Status of lymph node assessment was extracted from the surgical data. FIGO grading, staging, cervical invasion, lymph node involvement and lympho-vascular space invasion (LVSI) data were collected from the post-operative histology reports. Revised 2009 FIGO classification was used for staging. No personal identifiable data were collected, and strict confidentiality was maintained.

Data table was created using SPSS version 25 (IBM Corp., Armonk, NY, USA). Simple descriptive statistics were applied to get the results.

## Results

During the study period, 112 patients underwent surgery for endometrial cancer. This study included 90 patients with well-differentiated endometrial cancer (22 patients with poorly differentiated cancers were excluded) out of which pelvic lymph node dissection had been performed in 78 (86.7%). Among the 90 cases, 54 (60%) had metabolic risk factors.

Outcomes of surgical staging in differentiated endometrial cancer are summarized in Table 1. Cross-tabulation of lymph node status with risk factors for regional metastasis is shown in Table 2.

**Table 1:** Summary of surgical staging and post-operative histology-differentiated endometrial cancer. * Lymph node dissection has not been performed in 12 women ** Lympho-vascular space invasion data were not available in four cases

Surgical staging and postoperative histology	n (%) Yes	n (%) No
Pelvic lymph node dissection	78 (86.7)	12 (13.3)
Postoperative histology -Grade 1	77 (85.6)	Not applicable
Postoperative histology -Grade 2	13 (14.4)	
Lymph node involvement*	5 (5.6)	73 (81.1)
Cervical invasion	15 (16.7)	75 (83.3)
Lympho-vascular space invasion**	10 (11.1)	76 (84.4)

**Table 2:** Cross-tabulation of lymph node status and risk factors for invasion and metastasis.

Risk factors		Lymph node involvement		
		Yes	No	Dissection not performed
Lympho-vascular space invasion, n=10		3 (30)	6 (60)	1 (10)
Myometrial invasion, n=90	< 50%	1 (1.7)	48 (82.8)	9 (15.5)
	>50%	4(12.5)	25 (78.1)	3 (9.4)
Cervical invasion, n=15		3 (20)	10 (66.7)	2(13.3)

## Discussion

In our study, 35% (n=32) of patients had myometrial invasion of more than 50% of thickness. Out of these, 13.8% (4/29, three have not had lymph node assessment) of the patients with deep invasion were confirmed to have lymph node metastasis (Table 2) while there was only one patient (2%) with lymph node metastasis among those with superficial or no invasion. Therefore, in our study, superficial/no myometrial invasion has a negative predictive value of around 98% to exclude pelvic lymph node metastasis. Similar findings were obtained in another study, with pelvic lymph node metastasis of 15.2% and 17.1% among patients with deeply invasive Grade 1 and Grade 2 disease respectively [9]. Depth of myometrial invasion is one of the most important predictors of lymph node metastasis in addition to cellular differentiation [9]. Therefore, pre-operative pelvic MRI is used to triage patients for pelvic lymphadenectomy [4].

However, owing to lack of resources, many patients in Sri Lanka undergo surgery without preoperative MRI [8]. Consequently, it has been the local policy of the Apeksha Hospital Maharagama, Sri Lanka, to perform pelvic lymphadenectomy for patients with Grade 1/2 endometrioid endometrial cancer, who are referred without pre-operative pelvic MRI. In our sample, 86.7% (n=78) of the well-differentiated endometrial cancer patients underwent lymphadenectomy, out of which 6.4% (n=5) were positive for metastasis. Quite similarly, in a larger prospective study done in the USA, sub-analysis of the patients with FIGO 1 and FIGO 2 endometrioid endometrial cancer showed a pelvic lymph node metastasis rate of 8.0%.

Rising incidence of endometrial cancer could be attributed to increasing obesity and increased life expectancy [10-12]. In addition, unopposed endometrial estrogen exposure such as early menarche, late menopause, nulliparity, infertility, anovulation, polycystic ovary syndrome, and non-hormonal factors such as hypertension, diabetes mellitus and dyslipidemia are known to be associated with endometrial cancer [13,14]. This was reflected in the present study with 60% (n= 54) of the patients having at least one risk factor. It is worth noting the utility of these risk factors in triaging women with postmenopausal bleeding for endometrial biopsy, since those with risk factors would have a significantly higher probability of endometrial cancer than those without. Therefore, endometrial thickness should not be the only factor when decisions regarding endometrial biopsy are made [5,7].

One of the major advantages of performing surgical staging in well-differentiated endometrial cancer is to avoid unnecessary pelvic irradiation in patients with deeply invasive disease. Radiotherapy is often associated with both short-term and long-term complications [15]. In addition, in isolated pelvic recurrence of endometrial cancer, radiotherapy can be used as a treatment option with possible curative intent. Since re-irradiation is usually not performed in previously irradiated sites, this treatment option would not be available for a subcategory of patients with pelvic recurrence who have had prophylactic irradiation due to incomplete surgical staging (where post-operative histology reveals deep myometrial invasion and surgical lymph node staging has not been done).

Cervical involvement is also considered a risk factor for advanced disease [16,17]. Interestingly, 16.7% (n=15) of our patients had cervical involvement in the final pathological assessment. Traditionally radical hysterectomy (RH) was the practice where cervical stroma is involved. However, recent data from retrospective studies are suggestive of similar overall survival in both RH and simple hysterectomy plus adjuvant therapy [7]. Preoperative MRI could have picked up these patients before surgery henceforth allowing the options between simple hysterectomy followed by radiation or RH without radiotherapy. Even though this would not give any extra survival advantage, some patients might opt to undergo RH to avoid pelvic radiation which is associated with adverse effects including significant sexual dysfunction [15].

LVSI is considered as an independent risk factor for recurrence [6]. In our sample 11.1% (n=10) of the patients had LVSI. It was interesting that 33% (n=3, one did not have lymph node assessment) of the patients with LVSI were associated with lymph node metastasis while it was only 3% (n=2, nine did not have lymph node assessment) in those without LVSI. Another study done among early endometrioid endometrial cancer patients showed LVSI positively in 8.3% of the sample [18]. Due to these reasons, the European Society for Medical Oncology (ESMO) recommends adjuvant treatment for patients with LVSI [6].

Limitations

Single-center data and small sample size are limitations in this study. Our database only contained information in connection with the admission for primary surgical treatment and further follow-up data was not available. Therefore, this study could not comment on long-term adverse effects of lymphadenectomy (such as lymphedema, lymphocele formation) as well as survival outcomes. Multicenter long-term perspective studies would be required to assess disease-specific survival and complications of surgical staging of differentiated endometrial cancer. 

## Conclusions

Nearly one in seven patients with deeply invading differentiated endometrial cancer (FIGO Grade 1 and 2) had pelvic lymph node metastasis. In limited resource settings where preoperative pelvic MRI is not freely available, a policy of routine surgical pelvic lymph node assessment would be valuable in detecting stage IIIc disease. Furthermore, it would also avoid unnecessary prophylactic pelvic irradiation in patients with deeply invasive differentiated endometrial cancer, who have not had lymph node assessment. To assess survival outcomes and long-term benefits of this practice, multicenter prospective studies would be necessary.
